# Sex differences in COPD-related quadriceps muscle dysfunction and fibre abnormalities

**DOI:** 10.1177/1479973119843650

**Published:** 2019-05-26

**Authors:** Adithya Sharanya, Margherita Ciano, Shirmila Withana, Paul Richard Kemp, Michael Iain Polkey, Samantha Amanda Sathyapala

**Affiliations:** 1 Molecular Medicine, National Heart and Lung Institute, SAF Building, South Kensington Campus, Imperial College London, London, UK; 2 Respiratory Medicine, Royal Brompton and Harefield NHS Foundation Trust, Harefield Hospital, Hill End Road, Harefield, Middlesex, UK; 3 Respiratory Medicine, Royal Brompton and Harefield NHS Foundation Trust, Royal Brompton Hospital, First Floor, Fulham Road, London, UK

**Keywords:** COPD, gender, skeletal muscle, atrophy, muscle weakness, systemic inflammation

## Abstract

In chronic obstructive pulmonary disease (COPD), lower limb dysfunction is associated with reduced exercise capacity, increased hospitalizations and mortality. We investigated sex differences in the prevalence of quadriceps dysfunction and fibre abnormalities in a large COPD cohort, controlling for the normal sex differences in health. We compared existing data from 76 male and 38 female COPD patients where each variable was expressed as a function of gender-specific normal values (obtained from 16 male and 14 female controls). Female COPD patients had lower quadriceps muscle strength and peak workload on a maximal incremental cycle ergometry protocol compared to male patients. Female patients had a smaller type II fibre cross-sectional area (CSA) compared to male patients, suggesting a greater female preponderance to fibre atrophy, although this result was largely driven by a few male patients with a large type II fibre CSA. Female patients had significantly higher concentrations of a number of plasma pro-inflammatory cytokines including tumour necrosis factor alpha and interleukin 8 (IL8), but not lower levels of physical activity or arterial oxygenation, compared to males. Our data confirm results from a previous small study and suggest that female COPD patients have a greater prevalence of muscle wasting and weakness. Larger studies investigating sex differences in COPD-related muscle atrophy and weakness are needed, as the results will have implications for monitoring in clinical practice and for design of clinical trials evaluating novel muscle anabolic agents.

## Introduction

Chronic obstructive pulmonary disease (COPD) is a systemic disease.^1^ Lower limb muscle atrophy (wasting), weakness and loss of oxidative muscle fibres are associated with reduced exercise capacity^2,3^ and increased mortality in COPD patients.^4–6^ Sex differences in the risk of developing COPD are well described. Females are more likely to develop COPD as a result of tobacco exposure,^7^ plasma levels of inflammatory biomarkers in COPD patients differ between sexes^8^ and sex hormones play an important part in the pathogenesis of COPD.^9^ Only one small study has investigated sex differences in the prevalence of skeletal muscle dysfunction and abnormalities in COPD.^10^ Female patients had more evidence of muscle damage, less evidence of muscle regeneration and more muscle dysfunction than male patients. Sex differences in health, seen in the control group, were not accounted for, thus the reported differences between female and male COPD patients could simply have reflected normal gender differences.

We have previously collected clinical and quadriceps biopsy data from the largest single centre cohort of COPD patients.^2^ We reanalysed these data to determine whether there were sex differences in the prevalence of quadriceps muscle dysfunction and fibre abnormalities in COPD patients, over and above sex differences in our healthy age-matched controls, and if so, whether differences could be attributed to sex differences in potential aetiological factors.

## Methods

Data from our prior study^2^ were reanalysed. The original study was approved by the Royal Brompton and Harefield NHS Trust and Ealing and West London Mental Health Trust Research Ethics Committees (06/Q0404/35 and 06/Q0410/54), and participants gave written informed consent. The cohort consisted of 114 COPD patients (76 male and 38 female) and 30 healthy matched controls (16 male and 14 female). Exclusion criteria were diagnoses of heart, renal or liver failure; systemic inflammatory, metabolic or neuromuscular disorders; use of warfarin; or a moderate/severe exacerbation (i.e. requiring intervention) within the preceding 4 weeks. Post-bronchodilator spirometry, lung volumes (plethysmography), carbon monoxide diffusion capacity and resting arterialized capillary earlobe blood gas tensions were measured using previously described techniques.^11–13^ Fat-free mass index (FFMI) was calculated from bioelectrical impedance measurements (using the Bodystat 1500, UK) taken after the subject had rested for 20 minutes lying supine.^14^ A low FFMI was defined as an FFMI less than 15 kg/m^2^ in women and less than 16 kg/m^2^ in men according to previously published cut-offs in the work by Schols et al.^15^ Physical activity, that is, locomotion, standing, sitting and lying time over 12 hours on 2 days was measured using the DynaPort accelerometer (McRoberts BV, Netherlands), as previously described.^16^ Quadriceps strength was measured as supine isometric maximal voluntary contraction (MVC)^17^ in the dominant leg. Quadriceps endurance (T_80_) was assessed by time for force decline to 80% of initial force during repetitive magnetic femoral nerve stimulation in the dominant leg as described in the work by Swallow et al.^18^ Whole body exercise performance was assessed by a 6-minute walk test according to American Thoracic Society (ATS) guidelines^19^ and by symptom-limited incremental cycle ergometry with metabolic testing, as we have done previously.^20^ Percutaneous biopsy of the vastus lateralis of quadriceps was performed using the Bergstrom technique^21^ on an occasion separate from strength and cycle tests. Muscle samples were stored as previously described^2^ until cryosectioning. Detection of type I, I/IIa, IIa and IIx fibres in a sample of at least 100 fibres (to calculate fibre type proportions and median fibre cross-sectional area (CSA)) was done with immunohistochemistry as described in the work by Natanek et al.^2^

### Variables analysed

#### Patient demographics and COPD disease severity measures

Includes smoking history (pack-years), current medication, lung function (including FEV_1,_ TL_CO_), arterial partial pressures of oxygen and carbon dioxide, health-related quality of life (SF36 and SGRQ scores).

#### Body composition

Body mass index (BMI), fat-free mass (FFM) and fat-free mass index (FFMI, kg/m^2^), low or normal FFMI (low defined as <15 kg/m^2^ in females and <16 kg/m^2^ in males^22^), fat mass (FM, kg) and fat mass index (FMI, kg/m^2^).

#### Quadriceps function

Quadriceps strength measured as MVC and T_80_ (time in seconds for muscle force to drop from initial force (30% of MVC) to 80% of the initial force with repetitive magnetic stimulation of the femoral nerve).

#### Quadriceps fibre characteristics

Proportion of slow-twitch, oxidative type I; fast-twitch, oxidative/glycolytic type IIa; and fast-twitch, glycolytic type IIx fibres and median CSA per fibre type (µm^2^) as assessed by immunohistochemistry of frozen transverse muscle sections.

#### Whole body exercise performance and daily physical activity

Six-minute walk distance (6 MW, m) and peak oxygen consumption (VO_2_, ml/kg/min) from an incremental cycle ergometry protocol, expressed as a percentage predicted^23^ which corrects for sex. Time spent in locomotion and standing (minutes over 12 hours) and movement intensity in m/s^2^ from accelerometer readings over 2 days.

#### Selected markers of systemic and muscle inflammation

Plasma concentrations of a number of pro-inflammatory cytokines and the anti-inflammatory cytokine IL-10 and soluble tumour necrosis factor alpha receptors I and II (TNF-αRI and II) were available from luminex quantifications (performed by GSK laboratories, UK). Muscle NF-κB p65 and p50 and AP-1 c-Jun subunit bound to DNA were quantified by transcription factor assays run on nuclear extracts (Panomics lab, Fremont, California, USA). Muscle TNF-α transcript levels were quantified by qPCR.

### Normalization

Male and female controls were matched in terms of demographic data as previously reported.^2^ However, some measurements, for example height, were expected to differ by gender in normal subjects (see Online Supplemental Table E1). In those cases, raw data for each patient (for variables not already subjected to any normalization) were divided by the mean or median value (depending on distribution) for the same variable from controls of the same sex, to give ‘normalized values’. Where there were well-validated existing prediction equations that correct for sex, for example, differing equations that convert 6-minute walk distance,^24^ peak oxygen consumption on incremental cycle ergometry^23^ and quadriceps MVC^25^ to a % predicted for females and for males, these were used as a method of normalization, allowing % predicted for female and male COPD patients to be compared directly. This was because we considered this a more accurate and sophisticated correction than dividing by the mean or median value of the same sex control group. Certain variables, such as physical activity levels, would not be expected to have a systematic bias between healthy females and males, but variation between individuals as a result of other factors. For these variables, it was logical to compare absolute (not normalized) values between patients of different sexes.

### Statistical analysis

This was carried out using Microsoft® Excel 2011 and GraphPad Prism version 7.00 for Mac, GraphPad Software, La Jolla California USA, http://www.graphpad.com. Data distribution was determined through visual inspection of graphed data, D’Agostino and Pearson, Shapiro–Wilk and KS normality tests. Group differences were tested using unpaired *t*-tests or Mann–Whitney for normally and non-normally distributed data, respectively. Pearson’s correlation coefficient and *χ* ^2^ statistics were calculated to determine significance of linear correlations and differences in proportion. Level of significance was determined to 5% (*α* = 0.05). Comparisons of proportions were performed using Fisher’s exact test.

## Results

Demographics and clinical parameters in COPD patients and controls are shown in Table 1.

**Table 1.** Clinical characteristics of patients and controls split by sex.

Variable	Female controls (*n* = 14)	Female COPD (*n* = 38)	*p* Value	Male controls (*n* = 16)	Male COPD (*n* = 76)	*p* Value	*p* Value between controls	*p* Value between patients
Age (years)	66.5 (9.0)	65.5 (10.3)	ns	67.0 (2.2)	65 (11)	ns	ns	ns
Smoking history and lung function								
Pack-years	0.00 (8.1)	38 (22)	<0.0001	7 (38)	50 (36)	<0.0001	ns	0.0004
FEV_1_ % pred	110 (14)	44 (29)	<0.0001	108 (20)	37 (25)	<0.0001	ns	ns
TL_CO_ % pred	82 (20)	38 (22)	<0.0001	90 (14)	45 (28)	<0.0001	ns	ns
PaCO_2_ (kPa)	5.2 (0.8)	5.1 (0.9)	ns	5.3 (2.2)	5.1 (4.7)	<0.0001	<0.0001	ns
PaO_2_ (kPa)	10.9 (1.2)	9.1 (1.7)	<0.0001	11.5 (1.2)	9.3 (2.1)	0.0060	ns	ns
Body composition								
Height (cm)	164 (11)	161 (9)	ns	172 (11)	173 (23)	ns	0.0003	<0.0001
Weight (kg)	64 (17)	62 (22)	ns	76 (26)	73 (20)	ns	0.02	0.002
BMI (kg/m^2^)	24.7 (3.9)	23.6 (8.6)	ns	26.3 (4.9)	24.1 (4.8)	ns	ns	ns
% low BMI	0	3 (1/38)	ns	0	1 (1/76)	ns	ns	ns
FFMI (kg/m^2^)	15.5 (1.2)	14.6 (2.3)	ns	18.8 (3.9)	16.4 (2.5)	0.001	0.0003	<0.0001
% with low FFMI	21 (3/14) (3/14)	58 (22/38) (16/38)	0.029	6 (1/16) (1/16)	42 (32/76)	0.004	ns	ns
Quadriceps muscle function								
MVC (N)	298 (138)	196 (112)	0.0056	412 (119)	255 (130)	0.008	0.0015	<0.0001
T_80_ (s)	118 (81)	83 (58)	0.025	103 (112)	80 (25)	ns	ns	ns
Exercise performance and HRQOL								
6 MW (m)	600 (92)	384 (173)	<0.0001	618 (27)	389 (300)	<0.0001	ns	ns
6MW % pred	129 (9)	84 (29)	<0.0001	114 (24)	76 (56)	<0.0001	0.02	0.02
Peak VO_2_ (ml/kg/min)	19.9 (10.3)	10.9 (3.9)	<0.0001	11.9 (6.3)	24.2 (4.9)	<0.0001	ns	ns
Peak VO_2_ (% pred)	100 (35)	49 (18)	<0.0001	90 (30)	44 (21)	<0.0001	ns	0.05
Standing time (min)	298 (82)	229 (150)	0.018	219 (126)	161 (100)	0.04	0.02	0.007
Locomotion time (min)	91 (92)	40 (20)	0.001	95 (65)	41 (48)	<0.0001	ns	ns
SF36	90 (18)	49 (26)	<0.0001	84 (10)	52 (36)	<0.0001	ns	ns
SGRQ	2 (7)	55 (17)	<0.0001	4 (13)	54 (42)	<0.0001	ns	ns

BMI: body mass index; FFMI: fat-free mass index; MVC: maximal voluntary contraction; TwQ: twitch force; 6 MW: 6-minute walk distance; VO_2_: oxygen consumption on maximal incremental cycle ergometry; % pred: % predicted; SF36: Short Form 36; SGRQ: St George’s Respiratory Questionnaire; COPD: chronic obstructive pulmonary disease; Quadriceps endurance.

### Healthy controls

As expected, healthy males were taller and heavier and had greater muscle mass and quadriceps strength (MVC) than healthy females (Table 1). Minor fibre type proportion differences existed between the two sexes (Table 2), consistent with previous reports.^26^ Fibre CSA did not differ between sexes in the controls (Figure 2(c) and Table 2). Female controls had a 15% higher 6 MW % predicted (*p* = 0.02; Table 1) and spent significantly more time standing than the male controls (298 (82) minutes/12 hours vs. 219 (126) minutes/12 hours, *p* = 0.02; Figure 3(b)). Plasma interferon gamma (IFNγ) and TNF-α were the only markers of inflammation different in female than male controls, where levels were higher in females (*p* = 0.01 and 0.047, respectively).

**Table 2.** Fibre characteristics in quadriceps of COPD and controls, split by sex.^a^

Fibre type	Type I	Type I/IIa	Type IIa	Type IIx
Fibre type proportions (%)				
Control (*n* = 30)				
Females	57 (48)	3 (0)	40 (37)	0 (1)^b^
Males	48 (39)	1 (0)	45 (34)	4 (8)^b^
Patients (*n* = 114)				
Females	30 (18)	5 (1)	58 (53)	3 (5)^c^
Males	31 (23)	3 (1)	61 (53)	5 (9)^c^
Normalized patient values (*n* = 114)				
Females	0.5 (0.3)	2.4 (0.3)^c^	1.4 (0.9)	2.5 (0.8)
Males	0.6 (0.5)	0.9 (0.5)^c^	1.4 (0.8)	1.0 (0.4)
Median fibre cross-sectional area (µm^2^)				
Control (*n* = 30)				
Females	5120 (4570)	5220 (4520)	3440 (2870)	4660 (3900)
Males	5830 (5117)	5490 (4860)	4480 (4060)	4790 (4340)
Patients (*n* = 114)				
Females	4970 (3476)	4670 (3280)^c^	3300 (1610)^d^	2380 (1650)^e^
Males	5340 (4590)	5390 (4630)^c^	4370 (1570)^d^	3130 (2270)^e^
Normalized patient values (*n* = 114)				
Females	0.90 (0.50)	0.89 (0.47)	0.84 (0.41)^d^	0.49 (0.31)^c^
Males	0.89 (0.27)	0.93 (0.28)	0.89 (0.32)^d^	0.59 (0.34)^c^

COPD: chronic obstructive pulmonary disease.

^a^Values are median (interquartile range).

^b^ *p* ≤ 0.01: statistically significant differences between males and females within patient or within control group calculated with the Mann–Whitney *U* test.

^c^ *p* < 0.05: statistically significant differences between males and females within patient or within control group calculated with the Mann–Whitney *U* test.

^d^ *p* < 0.0001: statistically significant differences between males and females within patient or within control group calculated with the Mann–Whitney *U* test.

^e^ *p* ≤ 0.005: statistically significant differences between males and females within patient or within control group calculated with the Mann–Whitney *U* test.

### COPD patients

#### Lung function, smoking history and exacerbations

Male and female patients were matched in their lung disease severity, yet female patients reported less smoke exposure than male patients (37.5 vs. 50 pack-years, *p* = <0.0001). Three percent of both female and male patients had GOLD I COPD, 37% of females and 25% of males had GOLD II COPD, 37% of females and 34% of males had GOLD III COPD and 24% of females and 37% of males had GOLD IV COPD. There were no statistically significant differences between the percentages of females and males at each GOLD stage. The proportion of female and male patients in chronic respiratory failure (PaO_2_ <8kPa) were identical at 16% and of female and male patients using long term oxygen therapy (LTOT) were not significantly different (55% and 8%, respectively, *p* = 0.71, Online Supplemental Table E2). Thirteen percent (5/38) of the female and 21% (16/76) of the male patients were current smokers (ns). There were no significant sex differences in drug treatment (Online Supplemental Table E2), exacerbation number (1 (3) vs. 2 (3) exacerbations/year), nor proportion of frequent exacerbators ((≥2 per year), 42% vs. 29%, ns).

#### Body composition, quadriceps function and fibre characteristics

**Figure 1.**
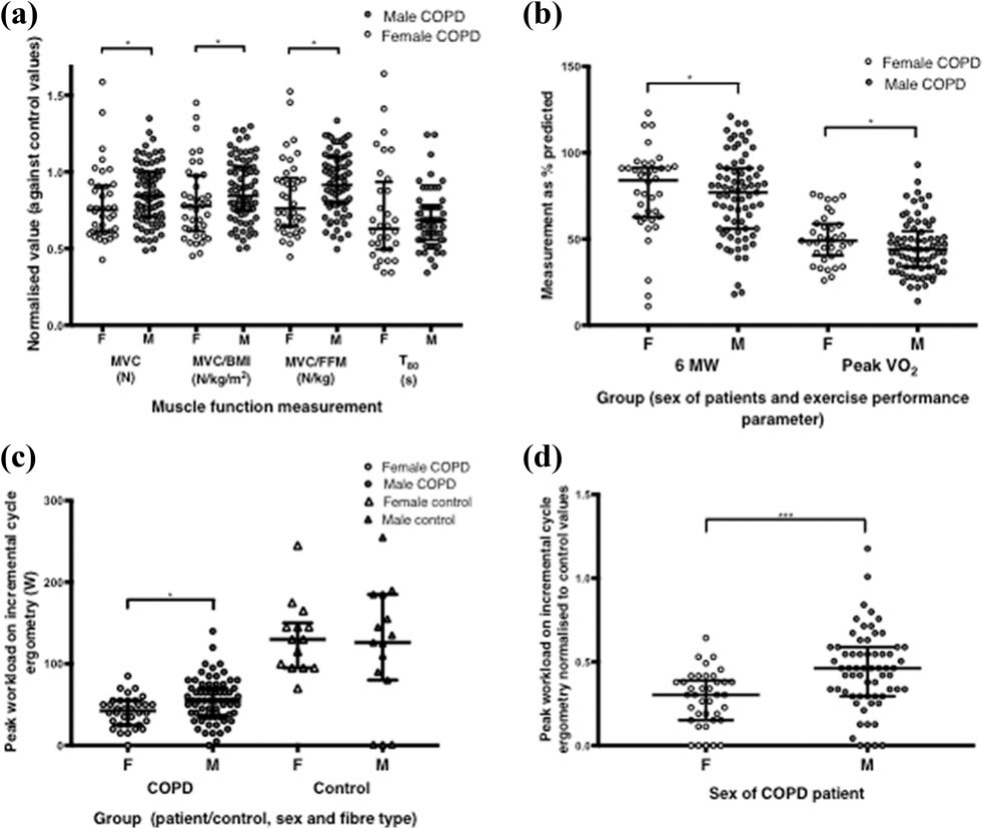
Muscle function and exercise performance in female and male COPD patients, and controls where appropriate. (a) Muscle function in female and male COPD patients, normalized for sex-specific control values. (b) Exercise performance in female and male COPD patients, as percentage predicted according to reference equations that include correction for sex. (c) Peak workload on maximal incremental cycle ergometry in female and male COPD patients and in female and male controls. (d) Peak workload on maximal incremental cycle ergometry in female and male COPD patients normalized for sex-specific values from controls. COPD: chronic obstructive pulmonary disease; MVC: maximal voluntary contraction; BMI: body mass index; FFM: fat-free mass; T_80_: quadriceps endurance.

Nutritional depletion, judged by a reduced FFMI, was prevalent in both female and male patients (58% and 42%, respectively, *p* = 0.12; Table 1), whereas a low BMI (<18 kg/m^2^) was less prevalent in both female and male patients (3% and 1%, respectively, *p* > 0.99; Table 1). FM and FMI were not significantly different between female and male patients (females 23.2 (13.9) kg vs. males 23.7 (10.3) kg, *p* = 0.94 and females 8.8 (5.2) vs. males 7.7 (3.0), *p* = 0.058, respectively). Absolute (not normalized) values of quadriceps strength (MVC), MVC/BMI and MVC/FFM were lower in female compared to male patients (196 (118) N vs. 255 (130) N, *p* < 0.0001 (see Table 1); 0.90 (0.42) vs. 1.28 (0.44), *p* < 0.0001; and 0.53 (0.22) vs. 0.67 (0.37), *p* = 0.0014, respectively). There was a significantly higher prevalence of an MVC/BMI ratio of <1.2, a known negative prognosticator in COPD,^5^ in female compared to male patients (82% in females vs. 32% in males, *p* < 0.0001). Similarly, normalized quadriceps MVC was lower in female compared to male patients 0.69 (0.39) vs. 0.83 (0.33), *p* = 0.04; Figure 1(a)), and normalized quadriceps MVC/BMI and MVC/FFM values (muscle strength per unit body/muscle mass) were both significantly lower in COPD females than COPD males (Figure 1(a)). Quadriceps MVC as % predicted, calculated using existing prediction equations from the work of Seymour et al.^25^ (hence corrected for sex), confirmed that quadriceps strength was lower in female compared to male patients (58 (26) % predicted vs. 74 (25) % predicted, *p* < 0.0001, Online Supplemental Figure E1). T_80_, both absolute and normalized, were not significantly different between female and male patients (85 (63) s vs. 80 (25), *p* = 0.32, and 0.55 (0.44) vs. 0.59 (0.24), *p* = 0.74).

**Figure 2.**
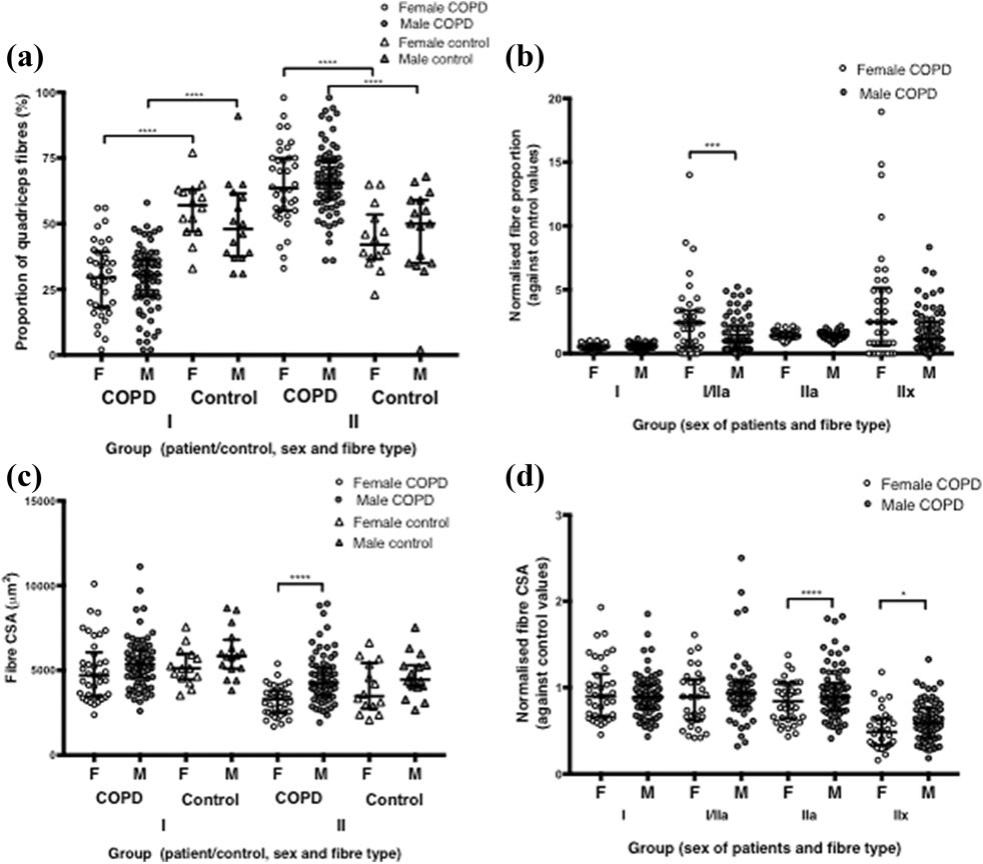
Quadriceps fibre characteristics in female and male COPD patients, and in controls where appropriate. (a) Quadriceps type I and type II fibre type proportions in COPD patients and controls. (b) Fibre type proportions in female and male COPD patients, normalized to sex-specific control values. (c) Quadriceps type I and type II fibre CSA in COPD patients and controls. (d) Quadriceps type I and type II fibre CSA in female and male COPD patients, normalized to sex-specific control values. CSA: cross-sectional areas; COPD: chronic obstructive pulmonary disease.

As described previously in this cohort, the COPD patients had a reduced type I fibre and an increased type II fibre proportion, compared to healthy age-matched controls^2^ (Figure 2(a) and Table 2). Prior to normalization, the only sex difference in fibre type proportions was that female patients had a slightly lower type IIx fibre proportion than male patients (3 (5) % vs. 5 (9) %, *p* = 0.037; Table 2), which is unlikely to be clinically significant. The normalized value comparison revealed that female patients had a slightly higher proportion of hybrid type I/IIa fibres compared to male patients (2 (3) vs. 1 (2), respectively, *p* = 0.04; Figure 2(b)), but no significant differences in any other fibre type. Prior to normalization, female patients had a significantly smaller CSA of IIa fibres compared to male patients (3300 (1610) vs. 4370 (1570) µm^2^, *p* = <0.0001). Female patients also had a smaller CSA of hybrid type I/IIa fibres (4670 (3280) vs. 5390 (4630) µm^2^, *p* = 0.003) and IIx fibres (2380 (1650) vs. 3130 (2270) µm^2^, *p* = 0.03) compared to male patients (Figure 2(c) and Table 2), although this was based on small numbers of fibres as I/IIa and IIx fibres were a minor proportion of total fibres. Female COPD patients also had a smaller normalized type IIa CSA (0.84 vs. 0.89 fold change, *p* = <0.0001) and type IIx fibre CSA (0.49 vs. 0.59 fold change, *p* = 0.03), and therefore smaller CSA of the type II fibre combined (0.84 vs. 0.90, *p* = 0.003) compared to male patients (Figure 2(d) and Table 2).

#### Exercise performance

Female patients achieved a lower absolute peak workload on maximal cycle ergometry than male patients (42 (26) W vs. 55 (35) W, *p* = 0.008; Figure 1(c)); a result that remained statistically significant when peak workload was normalized to sex-specific control values (0.3 (0.2) vs. 0.4 (0.3), *p* = 0.0007; Figure 1(d)). However, female COPD patients had a higher % predicted 6 MW performance and peak VO_2_ on maximal cycle ergometry compared to male patients (6 M W 84% predicted vs. 76% predicted, *p* = 0.02 and % peak VO_2_ 49% predicted vs. 44% predicted, *p* = 0.05; Figure 1(b)). There was a significantly higher proportion of female patients than male patients citing leg discomfort (rather than breathlessness) as the reason for stopping cycling (36% vs. 13%, *p* = 0.01).

#### Factors that may contribute to skeletal muscle dysfunction

##### C1 Resting blood gas tensions

There were no significant differences in resting arterial partial pressures of oxygen and carbon dioxide between male and female patients (Online Supplemental Figure E2A).

##### C2 Daily physical activity

Female COPD patients spent significantly more time standing than male patients (229 (150) minutes/12 hours vs. 161 (100) minutes/12 hours, *p* = 0.007; Online Supplemental Figure E2B), but the movement intensity was not significantly different between female and male patients (2.3 (11.7) m/s^2^ vs. 2.0 (6.9) m/s^2^). MVC/BMI, MVC/FFM and MVC as % predicted were correlated with locomotion time (but not standing time) in female patients (*r* = 0.40, *p* = 0.03; *r* = 0.44, *p* = 0.01; and *r* = 0.45, *p* = 0.01, respectively; Online Supplemental Figure E3A) but not in males (*r* = 0.1, *p* = 0.43 and *r* = 0.02, *p* = 0.83, respectively). There were no significant correlations between physical activity measures and fibre CSA in either female or male patient groups.

##### C3 Systemic levels of inflammation

**Figure 3.**
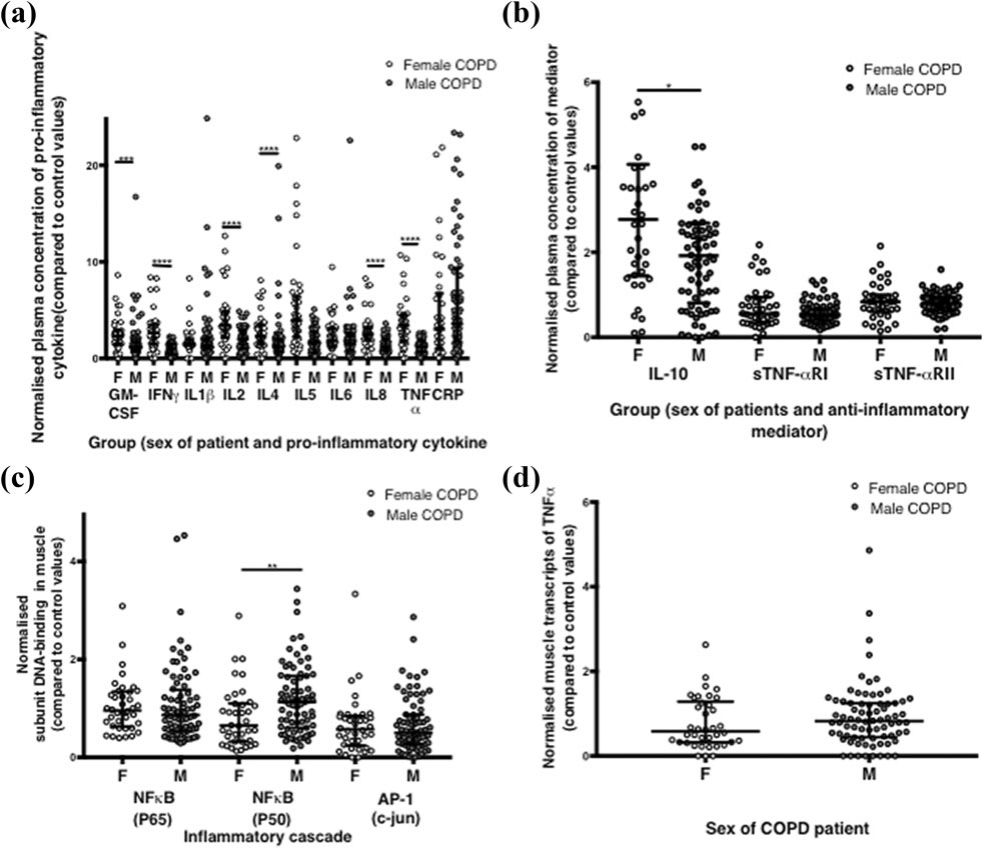
Systemic and muscle markers of inflammation in female and male COPD patients. (a) Levels of plasma pro-inflammatory cytokines in female and male COPD patients, normalized to sex-specific control values. (b) Levels of plasma anti-inflammatory markers in female and male COPD patients, normalized to sex-specific control values. (c) Markers of inflammatory cascades within muscle in female and male COPD patients, normalized to sex-specific control values. (d) Muscle levels of TNF-α mRNA in female and male COPD patients, normalized to sex-specific control values. COPD: chronic obstructive pulmonary disease. TNF-αRI: tumour necrosis factor alpha receptor I; IFNγ: interferon gamma; IL: interleukin; GMCSF: granulocyte monocyte colony stimulating factor; CRP: C-reactive protein; TNF-αRII: tumour necrosis factor alpha receptor II.

There were no significant differences in plasma pro-inflammatory cytokine levels between male and female COPD patients (data not shown). Since there were differences between healthy male and female in levels of systemic inflammation and activation of muscle inflammatory cascades (either statistically significant or trends), levels of inflammation in patients normalized for sex-specific values were compared. Female patients had significantly higher levels of normalized pro-inflammatory cytokines granulocyte monocyte colony stimulating factor, TNF-α, IFNγ and interleukins (IL) 2, 4, 5, 8, and anti-inflammatory IL-10 while there were no significant differences in IL-1β, IL6, C-reactive protein (CRP) levels or soluble TNF-αRI or RII (Figure 3(a) and (b) and Table 3). Fat mass and fat mass index were positively correlated with plasma CRP concentration in both females and males (females *r* = 0.46, *p* = 0.004, males *r* = 0.38, *p* = 0.01, and females *r* = 0.50, *p* = 0.01, males *r* = 0.40, *p* = 0.0004, respectively). There were no significant correlations between levels of pro-inflammatory cytokines and either MVC/BMI, MVC/FFM or type II fibre CSA in female patients. In male patients, there was a modest positive correlation between plasma CRP and type IIx fibre CSA (*r* = 0.38, *p* = 0.002; Online Supplemental Figure E3B).

**Table 3.** Levels of systemic inflammation values in male and female COPD patients normalized to sex-specific control values.

	Normalized values				
	Females		Males		
Systemic markers pro-inflammatory	Median	IQR	Median	IQR	*p* Value
IL-1β	1.5	0.6	1.3	1.1	0.13
IL-2	3.4	2.2	2.0	1.1	<0.0001
IL-4	4.0	1.5	1.2	0.7	<0.0001
IL-5	3.5	2.3	1.6	0.6	<0.0001
IL-8	2.5	2.0	1.3	0.8	<0.0001
GMCSF	2.3	1.6	1.2	0.7	<0.0001
TNF-α	3.4	1.9	1.3	0.7	<0.0001
IFNγ	3.0	1.5	1.0	0.4	<0.0001
CRP	3.0	5.8	3.6	7.6	0.19
Anti-inflammatory					
IL-10	2.8	1.5	2.0	0.9	0.03
Soluble TNF-αRI	0.55		0.52		0.37
Soluble TNF-αRII	0.84		0.80		0.87
Muscle markers	Normalized values				
TNF-α	0.6	1.0	0.8	0.8	0.25
NF-κB65	1.0	0.7	0.9	0.8	0.47
NF-κB50	0.7	0.8	1.1	1.1	0.004
AP-1	0.6	0.6	0.5	0.6	0.86

IL: interleukin; GMCSF: granulocyte monocyte colony stimulating factor; IFNγ: interferon gamma; TNF: tumour necrosis factor; AP-1: activator protein 1; CRP: C-reactive protein; TNF-αRII: tumour necrosis factor alpha receptor II; TNF-αRI: tumour necrosis factor alpha receptor I; COPD: chronic obstructive pulmonary disease.

##### C4 Muscle inflammation/inflammatory cascades

Absolute levels of muscle NF-κB p50, p65 or AP-1 DNA binding were not significantly different between female and male patients. However, COPD females had a significantly lower normalized muscle NF-κB p50 level than male patients (0.6 vs. 1.1, *p* = 0.003), but there were no significant differences in normalized muscle NF-κB p65 or AP-1 DNA binding (Figure 3(c)) nor muscle TNF-α mRNA levels between groups (Figure 3(d)). There was no correlation between muscle NF-κB p50 binding and fibre CSA in male patients.

## Discussion

### Summary of main findings

Female COPD patients have a greater prevalence of signs of type II fibre atrophy and loss of quadriceps strength compared to their male counterparts, above and beyond the normal sex differences in health. The finding of smaller type II fibres in female compared to male patients was, however, largely driven by the effect of three male COPD patients who had large type II fibre CSAs, even relative to male controls. Consistent with greater evidence of muscle fibre wasting and weakness, female patients achieved a lower peak workload on cycle ergometry than male patients, and a larger proportion of female patients cited leg muscle discomfort as the reason for stopping cycling compared with male patients. However, interestingly female patients had a greater percentage predicted 6 MW performance and peak oxygen consumption on cycle ergometry than their male counterparts. The reductions in muscle fibre size, muscle strength and peak workload occurred in female patients despite them having a lower pack-year smoking history and longer periods spent standing, which when carrying a load requires quadriceps activity, than male patients. However, this sex difference occurred in the presence of increased circulating pro-inflammatory cytokines in female compared to male patients (once normalized for sex differences in cytokines seen in the healthy controls). Although COPD females had evidence of a slightly increased early type I to type II fibre shift, there was no difference in the pure fibre type proportions nor T_80_. Muscle levels of NF-κB p50, relative to levels in their same sex counterparts, were lower in female than male patients.

### Comparison with existing literature on the subject

Our findings of female COPD patients demonstrating lower muscle strength and greater muscle atrophy are consistent with those of Ausin et al.^10^ However, with our larger sample size, we did not find that male patients had a greater type II fibre proportion as the previous study reports^10^ but data supporting a subtle increase in fibre type switch in females with COPD. Like the previous study, we found that female COPD patients have relative higher systemic inflammatory markers than males but we did not find direct correlations between increased markers of systemic inflammation and fibre atrophy, nor indeed between physical activity levels and fibre atrophy, in either sex. This suggests that any contribution of systemic inflammation or physical activity to fibre atrophy is moderated by other influences so that simple linear associations are not evident.

The reduction in peak workload on a maximal incremental cycle protocol in female compared to male patients is consistent with a previous report from Yquel et al.^27^ However, it is notable that we did not see the differences between male and female COPD patients in muscle fibre size and muscle strength translating to greater reductions in % predicted 6 MW and peak VO_2_ on cycle ergometry in the female patients. Published predictive equations have a high variability in their predictive power, suggesting that other factors not included in the calculations play an important role in the distance walked. It may be that using % predicted values introduces a sex difference not dependent on muscle function and mass but on other factors. However, with height (hence leg length), weight and lung function having such an effect on exercise performance using uncorrected values to compare male and female patients would have been less satisfactory. Furthermore, there are advantages of smaller fibre size to performance measured as peak oxygen consumption, namely smaller diffusion distance for oxygen,^28^ which may explain our results.

The canonical NF-κB pathway that signals in response to inflammation is dependent on heterodimers of p65 and p50 units translocating to the nucleus to induce transcription promoting skeletal muscle protein degradation^29^ and inhibiting myogenesis,^30^ at least with acute pathway activation. The fact that there were no differences in p60 DNA binding in males and females suggests that activation of the canonical NF-κB pathway in muscle is similar in males and females, despite the increased plasma levels of pro-inflammatory cytokines in female patients. The non-canonical NF-κB pathway associated with muscle disuse atrophy is dependent on p50 (and Bcl3) subunits not p65 subunits.^31^ There was evidence of increased p50 DNA binding, despite the fact there was less evidence of fibre atrophy, in male, compared to female, patients. Increased activation of the non-canonical NF-κB pathway is therefore unlikely to account for the greater prevalence of fibre atrophy seen in females. This also suggests that there are differences in activation of signalling mechanisms in the locomotor muscle of male and female COPD patients. The influence of oestrogens and menopause on skeletal muscle inflammation, damage and regeneration^32^ and differences in muscle protein synthesis/protein metabolism between sexes^33^ are well described and are good examples of how the biology of muscle damage and repair differs between the sexes. Sex hormones also modulate systemic inflammation; for example, androgens have been shown to suppress the immune system.^34^ Therefore, the sex differences we report could reflect differences in sex hormone levels between female and male COPD patients, which would support the use of androgenic agents, such as selective androgen receptor modulators, in the treatment of COPD-related muscle wasting, particularly in females.

### Study limitations

This is a cross-sectional study and therefore we are unable to comment on sex differences in rate of muscle fibre atrophy over time. It is possible, therefore, that the finding of more fibre atrophy and weakness in female patients was not a genuine sex difference, but that it was related to another factor, for example, that diffusion abnormalities develop earlier in female than male patients, that causes muscle atrophy and weakness. Although our sample size was large for a single cohort including biopsy data, there was still a relatively small number of patients, particularly females, and therefore it cannot be discounted that the sex differences relate to sampling bias of females who had greater evidence of fibre atrophy and weakness than the average female COPD population. Our control sample is also small and therefore may not be representative, although it is larger than the previous similar study and many other COPD muscle studies. The female controls had less cigarette exposure than the male controls which, if it affected the physiological variables we measured, may also have confounded genuine sex differences. For these reasons, we were careful only to normalize to control values where there was previous literature supporting the observed sex differences we found in our controls. Had there been measurements of thigh muscle CSA or another measurement of lower limb muscle mass, it would have been preferable to correct our strength data to one of these measurements, particularly as musculature versus adipose tissue distribution in the lower limb is likely to be different in females and males. We did replicate our findings of greater quadriceps weakness in female patients when MVC was converted to a % predicted value, using validated equations for expected values in females and males, although these equations again use global rather than lower limb muscle mass. Analyses of the minority fibre types in quadriceps, for example, hybrid I/IIa fibres and type IIx fibres were likely to be underpowered because of the small number of these fibres per subject. The multiple comparisons we made increased the possibility of false positive results; however, on the whole, our significant results were consistent using a number of similar parameters, for example, consistent between MVC, MVC/BMI, MVC/FFM and MVC % predicted giving us confidence that they were not random positive results. Had we measured circulating sex hormone levels in female and male patients, this would have been valuable to examine whether these could account for the observed sex differences and which, if any, physiological measurements correlated with androgen and oestrogen levels. From the age of our female patients, it is likely that the majority were postmenopausal; therefore our results cannot be assumed to generalize to comparisons of premenopausal female and age-matched male patients. It would have been useful to have had data on menopausal status and hormone replacement therapy (HRT) use from our female patients and controls.

### Implications for clinical practice

We have confirmed a previous report that female COPD patients appear more prone to quadriceps fibre atrophy and weakness than their male counterparts and identified that, in COPD, there are sex differences in aetiological factors and downstream signalling pathways that influence skeletal muscle structure and function. Further studies confirming sex differences in a larger patient cohort with a large comparative control population are required to see if these results hold, and whether the wasting and weakness in females is associated with androgen levels. These studies are necessary as the results would have implications for monitoring of patients in clinical practice, for stratification of participants in clinical trials of new treatments for COPD-related muscle atrophy, including new androgenic agents.

## Supplemental material

CRD-18-0071.R1_supplement_Natanek - Sex differences in COPD-related quadriceps muscle dysfunction and fibre abnormalities Click here for additional data file. CRD-18-0071.R1_supplement_Natanek for Sex differences in COPD-related quadriceps muscle dysfunction and fibre abnormalities by Adithya Sharanya, Margherita Ciano, Shirmila Withana, Paul Richard Kemp, Michael Iain Polkey and Samantha Amanda Sathyapala in Chronic Respiratory Disease
