# Supplementary material for: Sex differences in COPD-related quadriceps muscle dysfunction and fibre abnormalities
Source: Chron Respir Dis. 2019 May 26;16:1479973119843650. doi: 10.1177/1479973119843650 (PMC6537500; doi:10.1177/1479973119843650)
Supplement: CRD-18-0071.R1_supplement_Natanek - Sex differences in COPD-related quadriceps muscle dysfunction and fibre abnormalities [file CRD-18-0071.R1_supplement_Natanek.pdf]

SUPPLEMENTARY MATERIAL

CRD-18-0071.R1

Sex differences in COPD-related quadriceps muscle dysfunction and fiber abnormalities

Table E1: Variables predicted to be affected by sex and variables predicted not to be affected by sex

Variables predicted to be affected by sex	Variables predicted not to be affected by sex
Height	Body mass index (BMI)
Weight	
Fat free mass (FFM)	
Fat mass	
Fat free mass index (FFMI)	
Quadriceps strength (MVC)	Quadriceps endurance (T_{80})
Absolute measurements of exercise performance (e.g. 6MW distance in m, peak VO_2 in L/min)	% predicted values of exercise performance measurements (e.g. 6MW distance and peak VO_2 both as % predicted)
Peak workload (W_{max}) on cycle ergometry	
Quadriceps fibre type proportions	
Quadriceps fibre cross-sectional area (CSA)	

Table E2: Drug history of the COPD patients split by sex

Sex of patient	SABA	LABA	TIO	ICS	THEO	PRED	LTOT (>15 hr/day)	NIV
Female (n=38)	87	95	74	95	16	8	5	3
Male (n=76)	82	83	57	86	29	12	8	3

Values are percentages of patients prescribed each of the classes of medication, Abbreviations: SABA:short acting β agonist, LABA: long acting β agonist, TIO:tiotropium (long acting cholinergic antagonist), ICS: inhaled corticosteroids, THEO:theophylline, PRED:prednisolone, LTOT: long-term oxygen therapy, NIV: home nocturnal non-invasive ventilation. There were no statistically significant differences in medication usage between females and males, calculated with Fisher’s exact test.

FIGURES

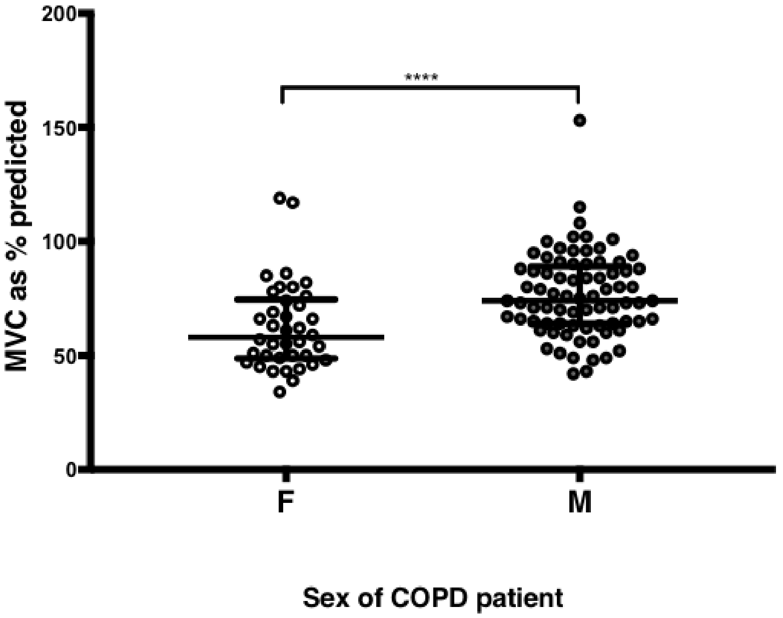

Figure E1 Quadriceps MVC as % of predicted value, using the equations from Seymour *et al*, in female and male COPD patients

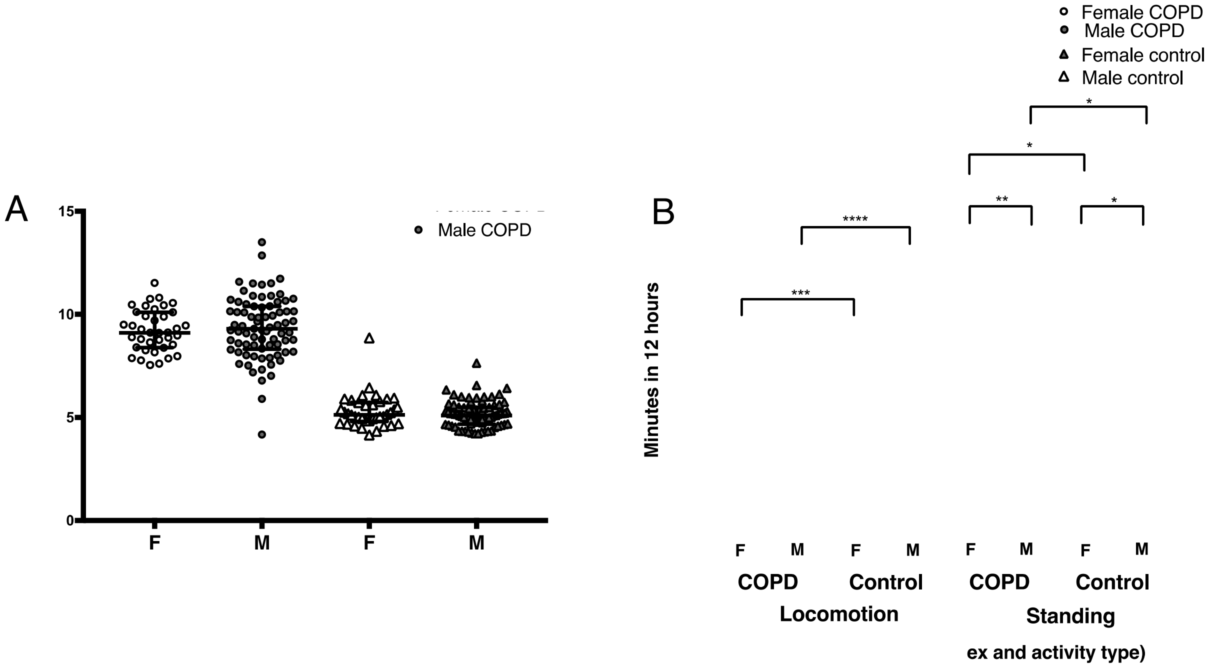

Figure E2 Arterial blood gas tensions in female and male COPD patients (A), and physical activity levels in female and male COPD patients and in female and male controls (B).

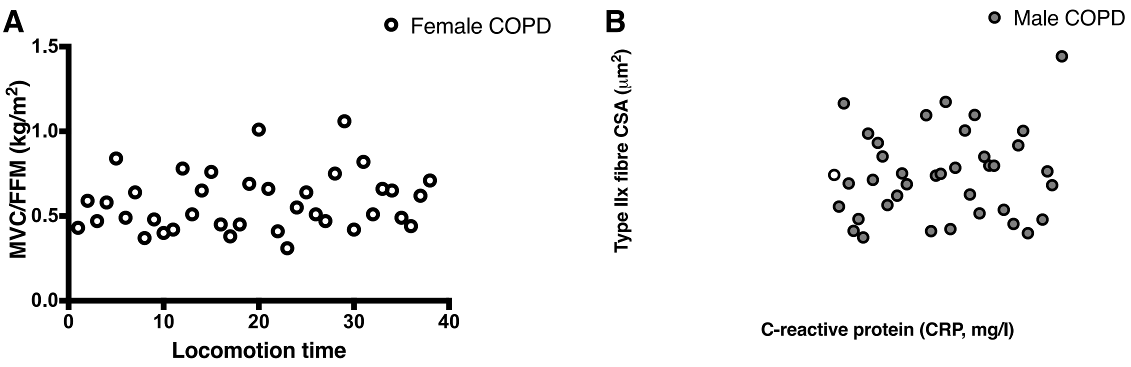

Figure E3: Scatter graphs of correlations between quadriceps strength and physical activity in female COPD patients (A) and between type 2 fibre CSA and plasma CRP concentrations in male COPD patients (B).

Abbreviations: MVC: maximal voluntary contraction, FFM: fat-free mass
